# Effects of Chronic Supplementation of L-Arginine on Physical Fitness in Water Polo Players

**DOI:** 10.1155/2021/6684568

**Published:** 2021-03-15

**Authors:** Jessica Gambardella, Antonella Fiordelisi, Luca Spigno, Lorenzo Boldrini, Giulia Lungonelli, Eugenio Di Vaia, Gaetano Santulli, Daniela Sorriento, Federica Andrea Cerasuolo, Valentina Trimarco, Guido Iaccarino

**Affiliations:** ^1^Department of Advanced Biomedical Sciences, Federico II University of Naples, Italy; ^2^Department of Medicine, Wilf Family Cardiovascular Research Institute, Einstein-Institute for Aging Research, Albert Einstein College of Medicine, New York, NY 10461, USA; ^3^Department of Molecular Pharmacology, Fleischer Institute for Diabetes and Metabolism (FIDAM), Einstein-Mount Sinai Diabetes Research Center (ES-DRC), Albert Einstein College of Medicine, New York, NY 10461, USA; ^4^Clinical and Sports Nutrition Service, Villa Montallegro Hospital, Genova, Italy; ^5^CIRIAPA Interdepartmental Center for Research on Arterial Hypertension and Associated Conditions, Federico II University of Naples, Italy; ^6^Department of Neurosciences, Federico II University of Naples, Italy

## Abstract

**Background:**

Ergogenic nutritional supplementation is sought by professional athletes for improving physical performance; nevertheless, scientific evidence to support the chronic use of L-Arginine among water polo players is missing.

**Methods:**

Seventeen male professional water polo players were randomly assigned to assume 5 grams per day of L-Arginine (*n* = 9) or placebo (*n* = 8) for 4 weeks. The players' fitness level was assessed in the maximal speed swimming test. Ear lobe blood samples taken before and after the effort for serum lactate content were analyzed. A speed-to-lactate ratio was generated at the baseline and after 4 weeks of treatment. We also tested the effects of L-Arginine *in vitro*, measuring NO production, mitochondrial respiration, and gene expression in human fibroblasts.

**Results:**

L-Arginine did not modify BMI, muscle strength, and maximal speed at 200 meters after 4 weeks. However, L-Arginine ameliorated oxidative metabolism to exercise as suggested by the statistically significant lower lactate-to-speed ratio, which was not observed in placebo-treated controls. *In vitro*, L-Arginine induced the expression of a key regulator of mitochondrial biogenesis (PGC1*α*) and genes encoding for complex I and increased the production of nitric oxide and the maximal oxygen consumption rate.

**Conclusions:**

Chronic L-Arginine is safe and effective in ameliorating the oxidative metabolism of professional water polo players, through a mechanism of enhanced mitochondrial function.

## 1. Introduction

The amino acid L-Arginine contributes to the regulation of energetic metabolism in cells, by participating in the Krebs cycle after conversion in citrulline [1]. In this reaction, the signaling molecule nitric oxide (NO) is also produced, which can participate in the contractility and metabolism of muscles, as well as in the potentiation of neuronal activity and immune response [2]. NO induces vasodilation and increases tissue perfusion, thus ameliorating the delivery of oxygen and nutrients for the production of ATP. Indeed, NO availability improves muscle mitochondrial respiration and biogenesis [3, 4].

Some studies associate these biological features of NO with the amelioration of muscle force production and endurance performance and recovery [5, 6]. Indeed, the nutritional supplementation of dietary nitrates in adults engaged in leisure activities has been associated with amelioration of the physical performance, as reported by reduced oxygen cost of submaximal exercise and resistance to high-intensity exercises [7, 8]. Interestingly, in elite athletes, the chronic effects of nitrate administration have never been investigated [9].

Professional water polo represents an interesting setup to study muscular and physical adaptations to mixed training characterized by high-intensity bouts alternated to lower intensity efforts [10].

Nutrition participates greatly in the development of endurance and power capabilities in response to training [11]. The use of products containing L-Arginine among athletes is diffuse, although the effects on performance are not clear and still controversial [12, 13]. The physiological concentrations of L-Arginine are generally sufficient to saturate endothelial nitric oxide synthase; nevertheless, acute effects of 6 grams of arginine have been demonstrated to increase the performance [14]. In particular, dietary supplementation of nitrates in recreationally active athletes leads to a reduction in the oxygen cost and improves tolerance to high-intensity exercise [15]. Interestingly, in elite athletes, the effects of nitrate supplementation are less evident, and almost all of the studies are concordant on the neutral effects on maximal performance [16]. It has to be noted, though, that most of the studies in elite athletes are referred to acute supplementation, and the effects of chronic supplementation of L-Arginine in elite athletes are far less explored.

The present study investigates the effects of 4-week, 5 grams per day, dietary L-Arginine supplementation on serum biochemistry and exercise performance in elite water polo players. Furthermore, in an *in vitro* model, we evaluated the effects of chronic exposure to increased L-Arginine concentration on parameters of mitochondrial biogenesis and function.

## 2. Methods

### 2.1. Water Polo Players

Seventeen male water polo players participating in the top-level division of the Italian Championship took part in the single-blind, placebo-controlled, parallel-group, randomized study. They were randomly assigned to the L-Arginine (Bioarginina Farmaceutici Damor®, *n* = 9) or placebo (*n* = 8) parallel groups. The anthropometric features are described in Table 1. Informed consent was obtained by all players before testing, and the experimental protocol was approved by the Ethical Committee of the Federico II University of Naples, Italy. The trial is registered in (NCT04626375).

### 2.2. Procedures

The players' fitness level was assessed in the maximal speed swimming test. After 10 min standardized warm-ups, participants swam 200 meters in an indoor, 25 m swimming pool at maximum speed. Ear lobe blood samples were taken before and after the effort and analyzed for serum lactate content using the reflectance photometric enzymatic reaction method (Accusport, Boehringer, Germany). A speed-to-lactate ratio was generated. Further assessment implied blood sampling for IGF1 and CK on serum and dynamometer measurements of upper limb maximal power.

The above-described assessments were performed at baseline and after 4 weeks of treatment with either placebo or a daily dose of 5 grams of L-Arginine *per os*. During the time of intervention, athletes trained daily according to the training schemes of the regular season. The placebo tablets were indistinguishable from those of L-Arginine as per shape and packaging. Therefore, the athletes were unaware of being on the treatment or placebo group.

### 2.3. Cells

Human Embryonic Kidney (HEK-293) fibroblasts were cultured in Dulbecco's minimal essential medium (DMEM) as previously described, 25 mM, and supplemented with 10% fetal bovine serum (FBS) at 37°C in 95% air-5% CO_2_. At 70% confluence, cells were treated with L-Arginine (a kind gift from Farmaceutici Damor, Italy) at a concentration of 500 *μ*M or vehicle (water), for 24 hours. After incubation, the cells were lysed or used for specific analysis.

### 2.4. NO Production

NO production was determined as previously described [17]. Briefly, HEK-293 were seeded in 24-well plates and incubated with 10 *μ*M DAF-FM Diacetate (Invitrogen) for 60 min at 37°C. After washing, the cells were incubated for an additional 15 min at room temperature to allow complete deesterification of the internalized probe. Raw fluorescence at 495/515 nm was registered every 10 sec using the Tecan Infinite 200 Pro plate reader. The fluorescence was corrected by the background signal derived from nonmarked cells.

### 2.5. Mitochondrial Respiration

Mitochondrial respiration was assessed as previously described [18], using the Seahorse Analyzer (Agilent Technologies, Santa Clara, CA, USA). We added carbonyl cyanide 4-(trifluoromethoxy)phenylhydrazone (FCCP, 0.5 *μ*M, Merck KGaA) and antimycin A (both 1 *μ*M, Merck KGaA) to each well. After each assay, cells were collected to quantify DNA using the QuantiFluor dsDNA System (Promega, Madison, WI, USA).

### 2.6. Real-Time PCR

Total RNA from HEK-293 cells was extracted using a TRIzol reagent (Invitrogen), and cDNA was synthesized using the ThermoScript RT-PCR System (Invitrogen), following the manufacturer's instruction. After reverse transcription reaction, real-time quantitative polymerase chain reaction (RT-qPCR) was performed with a SYBR Green real-time PCR master mix kit (Applied Biosystems, Foster City, CA, USA) as described [17] using StepOne instrument (Applied Biosystems). Primers for gene analysis are indicated in Table 2. All standards and samples were assayed in triplicate. Thermal cycling was initiated with an initial denaturation at 95°C for 5 min. After this initial step, 40 cycles of PCR were performed. Each RT-PCR cycle consisted of heating at 95°C for 15 seconds, 60°C for 30 seconds, and 72°C for 1 minute. The ratio of fold change was calculated using the Pfaffl method [19].

### 2.7. Statistical Analysis

According to the available literature [20], an average physical performance assessment based on the lactate-to-speed ratio for professional water polo players is around 7 with a standard deviation of 1.2. We calculated that to observe a 20% amelioration as effect of treatment, which is statistically significant with a power of 80% and alpha error of 10%, 7 volunteers per group are needed. Data are expressed as the mean ± standard error. Analysis of variance or Student's *t*-test was used as appropriate for continuous variables; chi-squared analysis was used to assess differences in expected distributions. *P* < 0.05 is considered statistically significant. All statistical analysis was performed on SPSS 24.0 (IBM-Italia, Segrate, Italy).

## 3. Results

### 3.1. *In Vivo* Studies

#### 3.1.1. Physical Performance

**Figure 1:**
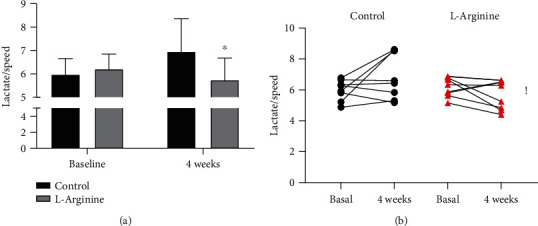
(a) Aerobic performance assessed as the serum lactate production to maximal speed on the 200 meters is similar between groups at the baseline, while significantly different after 4 weeks. (b) Evolution of lactate-to-speed ratio after 4 weeks in both groups. ^∗^*P* < 0.05 vs. control, ANOVA; ^!^*P* < 0.05 vs. Control, chi-squared test.

Athletes from the two groups at the baseline were similar by age, BMI, and muscle strength (Table 1). Baseline performance measured as the maximal speed at the 200 meters was not different between the control and the L-Arginine groups. Similar values of IGF-1, CK, and resting and exercise serum lactate were also observed between the two groups (Table 1). After 4 weeks, all the above parameters were not affected (Table 1). As previously established [20], the lactate-to-speed ratio represents a way to assess tolerance to exercise. While at the baseline, there was no difference between controls and L-Arginine athletes, after one month, the L-Arginine group presented a significantly lower lactate-to-speed ratio as compared to controls, suggesting a better oxidative metabolism to exercise (Figure 1(a)). This difference appears to be generated by the expected worsening of performance due to the intensive season training that was more frequent among the controls compared with the L-Arginine group (Figure 1(b)).

### 3.2. *In Vitro* Studies

#### 3.2.1. Mitochondrial Biogenesis

**Figure 2:**
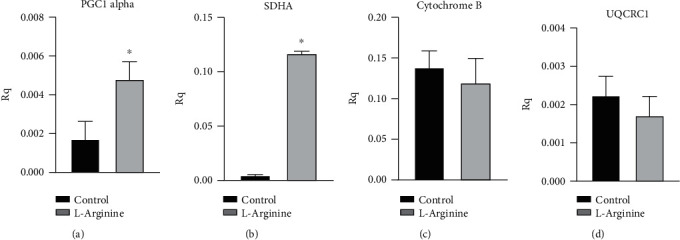
Gene expression in real time in L-Arginine vs. control-treated HEK-293 cells: (a) proliferator-activated receptor gamma coactivator 1*α*; (b) succinate dehydrogenase complex flavoprotein subunit A; (c) cytochrome B; (d) ubiquinol cytochrome c reductase complex I. ^∗^*P* < 0.05 vs. control.

To assess the changes in the energetic metabolism that are induced by chronic exposure to L-Arginine, we exposed fibroblasts for 24 h with the amino acid. This timing is enough to activate protein synthesis and therefore modify the cellular phenotype. L-Arginine induces the expression of the regulator of mitochondrial biogenesis (PGC1*α*) and genes encoding for complex I proteins such as Succinate Dehydrogenase A (SDHA), while genes of complex III (cytochrome B and ubiquinol cytochrome C1 reductase) were not affected (Figure 2).

#### 3.2.2. NO and Mitochondrial Function

**Figure 3:**
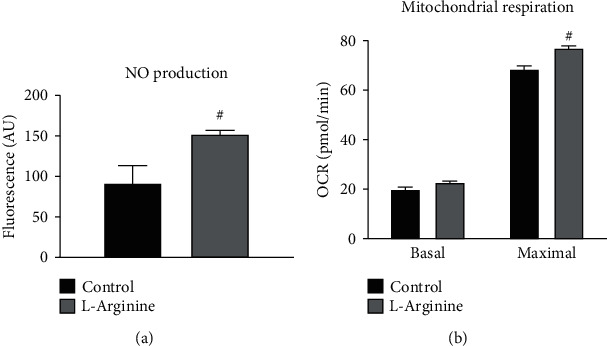
(a) Nitric oxide production in HEK-293 cells; (b) basal and maximal oxygen consumption rate (OCR) in HEK-293 cells. ^#^*P* < 0.05 vs. Control.

24 hr of L-Arginine induced a significant increase in the production of nitric oxide, which appeared to be almost double compared to control conditions (Figure 3(a)). Also, the maximal oxygen consumption rate, a measure of mitochondrial function, increased in L-Arginine-treated cells compared with control cells (Figure 3(b)).

## 4. Discussion

Our results provide the first evidence that in elite water polo players, chronic L-Arginine supplementation ameliorates aerobic energy expenditure, possibly due to a positive effect on mitochondrial energetics. Chronic L-Arginine administration was tested before as a tentative enhancer of physical performance. In fact, only one group reported an amelioration in physical performance in healthy-untrained subjects [21]. On the other hand, chronic L-Arginine supplementation did not show statistical effects on time to exhaustion in running and cycling in endurance well-trained athletes [13, 22]. In swimmers, using a different NO donor, the root beer juice, a similar finding was observed by Pinna et al. who showed that 7-day supplementation reduced the anaerobic threshold, without enhancement in maximum workload achieved and/or VO_2_max [23]. We confirm that we were unable to observe an amelioration of the physical performance of the examined athletes on average. Nevertheless, we verified that the physical response was achieved with lower production of lactates, thus suggesting the enhancement of the aerobic metabolism. Indeed, the *in vitro* study confirms the favorable effects of 24 hr exposure to L-Arginine on mitochondrial biogenesis and function.

Proteins and amino acids represent the most consumed ergogenic aids, with the esteem that at least one-third of athletes use them to maintain/potentiate athletic performance [11]. The consumption of amino acid supplements carrying vasodilatory properties is increasing in the sports field, due to the positive additional effect of vasodilation on muscle blood supply and athletic performance [24, 25]. In this scenario, the L-Arginine vasodilator effect has gained the favor of the athletes to improve their physical performance [14, 21, 26]. The perceived benefit of L-Arginine assumption, though, ailments the described arginine paradox, which has been described by Ignarro and coworkers; indeed, L-Arginine is a nonessential amino acid, synthesized in the small intestine from proline, glutamate, and glutamine, which is also abundant in diets with apparently no need for supplementation [27–29].

The vasodilator properties of L-Arginine reside in the fact that the amino acid is a substrate for nitric oxide synthases (NOS), a class of enzymes that share the ability to break arginine into citrulline and nitric oxide [30, 31]. Although its baseline plasma concentration is about 25- to 30-fold higher than the Michaelis-Menten constant Km of the isolated, purified endothelial NOS in vitro, further L-Arginine supplementation improves NO-mediated vascular function in vivo [32]. The possible mechanisms of action of L-Arginine supplementation might be identified in two main mechanisms. Indeed, being NO the most potent vasodilator produced in mammals, it has been shown to improve muscle performance by increasing blood flow, therefore inducing oxygen supplementation and carbon dioxide removal, and potentiation of mitochondrial biogenesis [33, 34]. The other possibility resides in the ability of citrulline to shift the energy metabolism to more anaerobic pathways and ameliorating mitochondria activity, through antioxidant properties [35]. Otherwise, L-Arginine has also been shown to stimulate the release of growth hormone (GH) [36, 37], which is a potent anabolic agent that favors cell growth and body energetics, which promotes muscle hypertrophy [38, 39]. Many effects of L-Arginine supplementation are also linked to improved carbohydrate oxidation and oxygen efficiency [40, 41], with reduced exercise-induced production of ammonia, lactate, fatty acids, and fat oxidation [42, 43].

In cells, we tested the ability of L-Arginine to interfere with energetic metabolism. We used L-Arginine to a *μ*M concentration and for a time that is adequate to observe transcriptional adaptation. The amelioration of the energetic metabolism we observed in this setup appears to be due to an increase in mitochondrial effectiveness, possibly related to the increased expression of proteins of the complex I of the electron transport chain. Further studies are warranted for a more extensive investigation of the mechanisms of L-Arginine to regulate mitochondrial gene upregulation, and the current study represents the appropriate background.

Water polo players represent a unique setup for the study of energetic metabolism adjustments in response to mixed endurance and power training. Water polo alternates aerobic-anaerobic metabolic demand [10]. During a water polo game, high-intensity efforts occur several times [44]; thus, muscle recovery and oxygen-dependent processes between efforts, such as phosphocreatine replenishment and the removal of accumulated intracellular inorganic phosphates, are crucial for performance [10]. Therefore, aerobic fitness is important in water polo, to be achieved with appropriate training and also with nutritional, especially ergogenic, strategies [45]. Indeed, previous studies had evaluated the possible role of *β*-alanine supplementation on physical fitness and performance, showing that this nutritional strategy may not be effective. Given the pleiotropic effects of L-Arginine, the possibility that this amino acid can ameliorate the physical performance of high-intensity athletes are manifold. Our data provide evidence that the performance evaluated as speed by lactate production is indeed ameliorated and suggest that an enhancement of mitochondrial efficiency due to upregulation of mitochondrial biogenesis could be the putative mechanism.

Our results imply that in high-intensity training, the nutritional ergogenic approach with L-Arginine can ameliorate the physical training responses.

## 5. Conclusions

The administration of chronic L-Arginine to high-intensity athletes is safe and effective in ameliorating physical performance. A mitochondrial mechanism can be evoked by chronic L-Arginine supplementation.

## Figures and Tables

**Table 1:** Anthropometric, physical, and biochemical characteristics of the study population.

	Baseline		4 weeks	
	Control	L-Arginine	Control	L-Arginine
Age (yrs)	29.3 ± 1.66	30.9 ± 1.31		
Height (cm)	191.5 ± 1.92	192.0 ± 1.78		
Weight (kg)	95.81 ± 2.19	100.7 ± 3.56	95.6 ± 2.22	100.0 ± 3.58
BMI (kg/m^2^)	26.1 ± 0.46	27.2 ± 0.56	26.07 ± 0.48	27.2 ± 0.56
CPK (*μ*L/L)	156.1 ± 25.1	170.0 ± 25.8	133.7 ± 13.1	184.0 ± 35.5
IGF1 (*μ*M/L)	327.9 ± 19.3	295.0 ± 19.0	226.5 ± 7.81	215.0 ± 10.0
Dynamometer (N)	76.1 ± 3.33	78.6 ± 2.02	76.12 ± 2.76	78.2 ± 1.88
Time to 200 mt (sec)	121.6 ± 1.62	123.0 ± 1.82	122.0 ± 1.93	124.0 ± 1.32
Basal lactates (*μ*U/L)	1.10 ± 0.10	1.04 ± 0.08	0.92 ± 0.08	0.83 ± 0.05
Postexercise lactates (*μ*U/L)	9.86 ± 0.41	10.0 ± 0.40	11.4 ± 1.22	9.25 ± 0.53

BMI: body mass index; CPK: creatine phosphokinase; IGF1: insulin-like growth factor.

**Table 2:** List of primers used in real-time PCR.

Gene	Sense primer sequence	Antisense primer sequence
18 S	5′-GTAACCCGTTGAACCCCATT-3′	5′-CCATCCAATCGGTAGTAGCG-3′
Cyt B	5′-CCTAGGCGACCCAGACAATTAT-3′	5′-TCATTCGGGCTTGATGTGG-3′
SDHA	5′-CATACTGTTGCAGCAGCACAGG-3′	5′-CCACCAAATGCACGCTGATA-3′
UQCRC I	5′-CCTACGCACTCGAGAGCAC-3′	5′-AGGTGTGCCCTGGAATGCTG-3′
PGC-1*α*	5′-AAACTTGCTAGCGGTCCTCA-3′	5′-TGGCTGGTGCCAGTAAGAG-3′

Cyt B: cytochrome B; SDHA: succinate dehydrogenase complex flavoprotein subunit A; UQCRC I: ubiquinol cytochrome c reductase complex I; PGC-1*α*: proliferator-activated receptor gamma coactivator 1*α*.

## Data Availability

Data are available at 10.6084/m9.figshare.13489977.
